# Suicide in rural agrarian culture: revealing the micro dimensions of suicidal behavior in Gunungkidul Regency, Indonesia

**DOI:** 10.3389/fsoc.2025.1588593

**Published:** 2025-10-17

**Authors:** Sunit Agus Tri Cahyono, Eko Wahyono, Ikawati Ikawati, Akhmad Purnama, Tateki Yoga Tursilarini, Sri Yuni Murtiwidayanti, Andayani Listyawati, Trilaksmi Udiati, Suryani Suryani, Tri Gutomo, A. Nururrochman Hidayatulloh, Irmawan Irmawan, Rizal Akbar Aldyan, Hari Harjanto Setiawan

**Affiliations:** National Research and Innovation Agency (BRIN), Jakarta, Indonesia

**Keywords:** rural, suicide, micro dimension, agrarian culture, community resilience

## Abstract

**Background:**

Suicide is a serious and complex global public health problem, affecting individuals from diverse social backgrounds, genders, and ages. Gunungkidul Regency in the Special Region of Yogyakarta, Indonesia, has the highest suicide rate in the region, making it a crucial location for understanding the contributing factors to suicide in a rural, agrarian cultural context.

**Methods:**

This study employed a qualitative descriptive design with a naturalistic approach to understand the meaning of individuals’ lived experiences in their natural context without researcher intervention. Data were collected through semi-structured interviews with 21 participants, consisting of 15 family members of individuals who died by suicide and six community stakeholders (community leaders, religious leaders, health workers, NGO representatives, local police officers, and village administrators). All interviews were audio-recorded, transcribed verbatim, and analyzed using an inductive thematic approach. Two researchers conducted coding independently, with validation through peer discussions, triangulation of interview data, field notes, and document review to ensure the credibility of the findings.

**Results:**

The analysis revealed two main patterns of suicide: altruistic suicide, associated with strong social cohesion, found in Playen, Wonosari, and Karangmojo sub-districts; and egoistic suicide, reflecting weak social ties, found in Semin and Ngawen. Contributing micro-factors include depression, economic stress, and chronic illness, which mutually exacerbate an individual’s psychological state. Seasonal stress, such as crop failure during a prolonged dry season, increases the risk. In local culture, suicide is sometimes interpreted as a form of “rebellion” against religious and social norms.

**Conclusion:**

These findings demonstrate the importance of early detection of mental health disorders, community economic empowerment through cooperatives, and culturally based education to address myths and stigma surrounding suicide. Prevention efforts must consider agrarian communities’ social and cultural context more deeply.

## 1 Introduction

Death by suicide is an act of a person or group as an escape from reality. The motive for suicide is someone’s thoughts about ending their life (Gonçalves et al., 2014) and is associated with suicidal ideation (Reynolds, 1991). Factors that cause suicide include acts of domestic violence, injustice in love towards children, romantic relationships, and disharmonious relationships between siblings (Setiadi et al., 2022). This action is carried out by farmers individually and collectively (Hayden et al., 2022). Social inequality and poverty in rural areas are essential in increasing rural farmer suicides (Sai Sravanth and Sundaram, 2019). Economic factors are the most common risk factors for suicide (Arafat et al., 2021).

Suicide is a global epidemiological problem regardless of social status, gender, and age (Sun et al., 2020; Zhou et al., 2023). Data sourced from the World Health Organization (WHO) states that the number of deaths due to suicide each year reaches 800,000 people, or one death every 40 s (WHO, 2019). In Japan, jouhatsu culture is based on a culture of shame that a person suffers from, which stimulates suicidal behavior. This action started from a serious problem that gave birth to depression (Dong et al., 2018; Kim and Lee, 2022), frustration (Fagbenro et al., 2019), the epidemic of a disease (Maatouk et al., 2022), and ended with the individual’s suicide. The five countries with the highest suicide index, namely Lesotho, 72.4; Guyana, 40.3; Eswatini, 29.4; South Korea, 28.6; and Kiribati, 28.3 per 100,000 people (Suicide Rate by Country 2023, 2023). In Southeast Asia, the suicide rate (17.3) per 100,000 people is higher than the global average (4.3). WHO targets reducing the death rate due to suicide below 3.4 as one of the goals of the SDGs mental health action plan for 2013–2030.

Farmer suicides are a global phenomenon, often linked to economic and social pressures (Murugan and Sivagnanam, 2018). Deaths in Indonesia due to suicide in 2018 were 3.4 people per 100,000 population. In terms of gender, it consists of 4.8 men and 2.0 women. In Gunungkidul Regency, which has the highest prevalence in the Special Region of Yogyakarta, 176 suicides were reported between 2018 and 2023, with economic hardship and health issues being major contributing factors (Nurdiyanto and Jaroah, 2020).

Individual psychological factors are related to social and cultural factors that lead a person to suicidal behavior. The Three Step Theory (3ST) explains: (1) Suicidal ideation results from the interaction of hopelessness and psychological pain, (2) this interaction is the main protective factor against suicidal ideation, (3) the idea of developing into a suicide attempt is facilitated by dispositional, contributory, and practice to commit suicide (David Klonsky and May, 2015). In Emile Durkheim’s thinking, suicide occurs because of social facts and differences in social currents that arise in a society; the variables of integration and regulation are included as indicators of the closeness between individuals and society (Aguilar, 2022). Drawing on Durkheim’s theory, this research categorizes suicides into altruistic and egoistic types.

Bronfenbrenner’s ecological theory explains that five systems influence individual development: microsystem, mesosystem, exosystem, macrosystem, and chronosystem (Adu and Oudshoorn, 2020; Arifin and Teh, 2020). Suicide cases are caused by micro dimensions, including psychological factors (Courtet et al., 2020; Hom et al., 2019; Mamun et al., 2020), major depression, mental health problems, individual psychology, economics, and family. The rise of suicide cases in rural areas, which have a strong culture of kinship and local wisdom, is a concern. The overall suicide rate in rural areas is higher than in urban areas. On a micro-scale, in agricultural areas, there is depression, psychological pressure (Thai et al., 2020), mental health, and economic issues in villages (Son et al., 2020).

The causes of the mezzo scale include stigma, pressure, and social isolation (Zhang, 2019), bullying (Cuesta et al., 2021; Goulah-Pabst, 2021), inequality, discrimination, and oppression (Hochhauser et al., 2020). On a macro scale, suicide can start from a decrease in the level of social integration due to unemployment (Mattei and Pistoresi, 2019; Men et al., 2022), economic crisis (Demirci et al., 2020), and disease pandemics (Sripad et al., 2021). This study explores the phenomenon of suicide in the agrarian community of Gunungkidul Regency, Yogyakarta, Indonesia. It also explains the factors contributing to suicide in the region at the micro level, namely psychological, economic, and physical health aspects. The novelty of this study is that it addresses a substantial and specific issue through a case study approach of suicide in a rural agrarian culture. Until now, most people have assumed that suicide occurs mostly in urban areas.

## 2 Methods

### 2.1 Study design and participants

This research employs a qualitative descriptive approach, employing a collective case study design within a naturalistic inquiry framework (Bradshaw et al., 2017; Lam et al., 2019). The collective case study design explores cases in Gunungkidul Regency to capture the complexity of experiences from various families and community stakeholders. The qualitative descriptive approach was chosen to generate direct descriptions of informants’ experiences. At the same time, the collective case study design allows for comparisons across multiple cases in various sub-districts in Gunungkidul. The naturalistic framework guides researchers in studying suicide within the informants’ everyday contexts without manipulation, to capture their lived realities. Therefore, this research is oriented towards understanding how informants interpret the social and cultural dynamics surrounding suicide. Data were collected through semi-structured interviews, observations, and document reviews.

### 2.2 Data collection

The research location was purposively selected (Andrade, 2020; Campbell et al., 2020) in Kunungkidul Regency because this area has the highest suicide data compared to other regencies in the Special Region of Yogyakarta (DIY) Province. Interviews were conducted with informants available for contact during the research period. Informants were selected purposively based on relevance to the research questions, accessibility, and availability. A total of 21 informants were interviewed, consisting of 15 family members of individuals who died by suicide and six key stakeholders from related sectors (community leaders, religious leaders, Health Service officers, representatives of non-governmental organizations (NGOs), members of the sub-district police, and members of the Village Development Army). The participating families came from five sub-districts in Gunungkidul Regency: Ngawen, Playen, Semin, Karangmojo, and Wonosari.

Data collection techniques included in-depth interviews with selected informants, observation, and document review. The research team collaboratively developed a semi-structured interview guide, utilizing relevant literature and the research objectives to ensure comprehensive coverage of key thematic domains related to suicide in rural agrarian communities. Each item was formulated to generate an in-depth narrative, considering the cultural context of Gunungkidul Regency. Before data collection, the guide underwent an internal peer review within the research team to assess its validity, clarity, and alignment with the research objectives.

To ensure reliability, all researchers who served as interviewers received training in qualitative interview techniques, ethical considerations, and scientific writing standards. A pilot test was conducted with selected participants from non-sample communities to refine the question structure, sequence, and probing strategies. Final verification of the guide was achieved through iterative revisions based on feedback from the pilot test phase, methodological discussions within the research team, and the application of triangulation principles during subsequent data collection and analysis.

All researchers, holding doctoral and master’s degrees, conducted interviews by dividing the informants into groups using a collaboratively developed guideline. The interview process took gender into account to ensure a comfortable environment. All researchers received training in standard methodology and scientific writing techniques. The researchers did not know the informants at the time of the study, but they shared a common cultural background. All informants understood the research objectives because they had been informed about the research before the interviews.

Each key informant participated in one to three in-depth interview sessions, depending on the richness and complexity of the information provided. Individual sessions lasted approximately 60 to 120 min, facilitating rapport building, exploring sensitive topics, and comprehensive inquiry. Follow-up sessions were arranged when clarification or elaboration of emerging themes was needed. Interviews with each informant continued until no new substantive themes emerged and responses began to recur among participants, signaling data saturation. Saturation was assessed iteratively throughout data collection by cross-checking transcripts and field notes among the research team. The researchers decided to conclude additional sessions collectively to ensure complete thematic coverage and robustness of the findings.

While the overall thematic framework of the semi-structured interview guide remained consistent across all participants, the content and emphasis of specific questions were tailored to the roles and experiences of each informant group. For the 15 family members of suicide victims, questions prioritized personal narratives, familial relationships, perceived causes, and the sociocultural context surrounding the suicide. For the six community informants, consisting of community leaders, religious leaders, health workers, NGO representatives, sub-district police officers, and members of the Village Guidance Team, interview topics emphasized community-level perceptions, prevention strategies, institutional responses, and broader socioeconomic and cultural dynamics. This differentiation ensured that the data captured experiences at both the individual level and structural or community perspectives, enabling a comprehensive understanding of the phenomenon while maintaining comparability across thematic domains.

### 2.3 Data analysis

Data were analyzed using a qualitative descriptive approach within a naturalistic framework, emphasizing describing participants’ experiences in their real-world context (Miles et al., 2018). The analysis proceeded through three iterative stages: (1) data reduction, involving careful reading and coding of interview transcripts; (2) data display, where emerging codes and categories were organized into thematic matrices; and (3) conclusion drawing and verification, in which themes were refined and validated across sources. To enhance credibility, two research team members independently coded the transcripts. Coding was conducted inductively, allowing themes to emerge from the data rather than being imposed *a priori*. Differences in coding were discussed in peer debriefings until consensus was reached. Triangulation across family members and community stakeholders, as well as comparison with field notes and documents, further strengthened the trustworthiness of the findings (Hoque et al., 2013).

### 2.4 Trustworthiness and ethics

This study used four criteria to improve research quality during data collection and analysis: credibility, transferability, dependability, and confirmability (Krefting, 1991). The credibility strategy utilised triangulation and peer review techniques. The transferability strategy ensured that other researchers could understand the study by producing a detailed, clear, systematic, and reliable report. The dependability strategy was implemented through a dependability audit conducted by the supervisor. If the research results are a function of the research process, then this study meets the confirmability standards.

The Indonesian National Agency for Research and Innovation (BRIN) approved this study. Throughout the research process, special measures were taken to ensure the safety of the informants, who were considered a highly sensitive population due to the prevalence of suicide in their families. Participants were given pseudonyms for privacy. All participants gave verbal consent before being interviewed and recorded. This consent was an ongoing negotiation with the participants, as qualitative research is suited to complex and changing environments. Life story interviews were conducted in a comfortable location for the informants, and they were allowed to terminate the interview at any time.

## 3 Results

Based on field data and interviews with families and relevant parties, it was found that the suicide phenomenon in the Gunungkidul agrarian community has complex dimensions. This research also uncovered an empirical phenomenon that is little known to many (Colorafi and Evans, 2016). Families consider suicide a taboo topic. The following sub-chapter outlines these findings within the context of the local social and economic life.

### 3.1 The phenomenon of suicide in an agricultural society

Gunungkidul Regency comprises 18 sub-districts, 144 villages, 1,416 hamlets, 1,583 RWs (community groups), and 6,844 RTs (neighborhood units). Most of the population of this district lives in rural areas with an agrarian culture, and their livelihoods are in the agricultural sector. Interview results indicate that suicide risk factors are not single, but multidimensional, encompassing psychological aspects (such as depression and loneliness), economic (poverty, crop failure), social (family conflict, isolation), and cultural and spiritual factors. Some factors are systemic and interconnected, demonstrating the need for a cross-sectoral approach to prevention efforts. The following table presents the demographic characteristics of key informants along with the main suicide risk factors they reported based on their individual experiences and observations (Table 1).

**Table 1:** Demographic characteristics and suicidal risk factors of key informants.

Participant group	*n*	Gender (M/F)	Age range (years)	Occupation	Relationship to suicide case	Main reported suicidal risk factors*
Family members of suicide victims	15	9 / 6	36–91	Farmer (majority), homemaker, student	Parent, spouse, sibling, or child of the victim	Depression, economic hardship, chronic illness, loneliness, family conflict, rejection of a marriage proposal, failure to pursue spiritual knowledge
Community leaders	2	2 / 0	45–60	Village head, community leader	Not directly related	Economic instability, prolonged dry season, crop failure, and weak social integration
Religious leaders	1	1 / 0	50–55	Religious leader	Not directly related	Religious and cultural myths (pulung gantung), stigma around mental illness
Health service personnel	1	0 / 1	40–45	Public health officer	Not directly related	Limited mental health services, lack of early detection
NGO representative	1	0 / 1	35–40	NGO staff	Not directly related	Poverty, the absence of preventive programs, and low community awareness
Sub-district police	1	1 / 0	40–50	Police officer	Not directly related	Social isolation, family neglect
Village Guidance Army	1	1 / 0	40–45	Military personnel	Not directly related	Weak family support, economic burden

*Risk factors are based on aggregated qualitative reports from each informant group, not individual diagnostic assessments.

**Figure 1:**
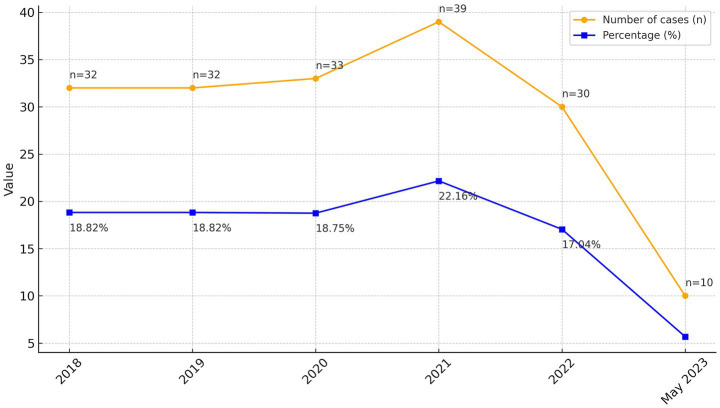
Suicide cases by year in Gunungkidul Regency (2018–May 2023).

As a result of this farm style, farming communities are characterized by a collective consciousness and strong family ties united in farming culture. These agricultural norms serve as guidelines for helping each other overcome various social welfare problems and establishing a culture of harmony in the lives of village communities. Gunungkidul’s agrarian culture emphasizes collective consciousness and strong kinship ties. However, economic instability and social changes have eroded these values, increasing vulnerability to suicide (Figure 1).

The problem of deaths due to suicide in Gunungkidul Regency has not been resolved. Data sourced from the Gunungkidul Health Service in 2023, cumulatively over five years, 176 cases of suicide were found. A total of 97 cases occurred from April 2018 to April 2020. In 2021, 39 cases were found. In 2022, there were 30, and finally, until the beginning of May 2023, 10 cases of suicide were reported again. Suppose socioeconomic conditions measure community status using education, employment, and income indicators. In that case, the majority of the social group of the families of suicide perpetrators is classified as of low economic status. More details can be seen in the following Table 2.

**Table 2:** Risk factors for suicide in Gunungkidul Regency (health service data).

Year/Period	Total cases (n)	Psychological factors*	Economic factors**	Physical health factors***	Other reported factors****
April 2018 – April 2020	97	Depression, hopelessness, loneliness	Economic hardship, crop failure	Chronic illness	Family conflict
2021	39	31 cases (79.49%) due to micro factors: 27 depression/economic/chronic illness (87.18%), two mental disorders (5.12%), two loneliness after spouse left (5.12%)	Debt, unemployment	Disability	Rejection of marriage, failure to pursue spiritual knowledge
2022	30	Depression, hopelessness	Reduced income, crop failure	Chronic illness	Cultural beliefs (pulung gantung)
January – May 2023	10	Similar patterns to previous years	Similar patterns to previous years	Similar patterns to previous years	—

Attempts to take one’s own life without warning are carried out when the perpetrator is alone or when family members are careless. Empirical conditions show that of the 39 suicide cases in Gunungkidul in 2021, 31 people were due to micro factors (79.49%), including 27 people (87.18%) identified as committing suicide due to depression, economic problems, and chronic illness. Until you feel tired and hopeless. Two people (5.12%) indicated suicide due to mental disorders in residents. Two people (5.12%) felt lonely and isolated from their families because their spouses left them.

Occupations that have a higher suicide rate are farmers/farm laborers (32 people or 82.05%), followed, respectively, by household work (3 people/7.69%), students (2 people/5.13%), fishermen and casual labourers, each one person (2.56%). Most of the suicide methods were by hanging oneself (97.44%), the remainder by drinking poison (2.56%). Thirty-two people (82.05%) used plastic ropes, ropes, and slings to commit suicide; one person used a belt (2.56%); Sarongs, strings, and scarves were used by five people (12.82%); and one person used poison (2.56%). The place of suicide was at home for 22 people (56.41%); a Tree for eight people (20.51%); an Animal cage for six people (15.38%); 1 person’s hut (2.56%); a Farm owned by one person (2.56%); and a drying area for one person (2.56%).

Concerning gender, it is noted that there is a gender gap, where in 2021, there were almost twice as many suicide cases among men as women, namely 25 people (64.10%) versus 14 people (36.15%). Most of the suicide cases at the research location occurred in the elderly in the age range 60–91 years, 28 people (71.79%), with details of 19 men and nine women. The remaining nine adults were between 36 and 57 (23.07%), and two were young people (5.14%). The data above shows that there is a tendency that men over 60 years of age are more likely to commit suicide compared to those under age due to various exposures beyond micro factors.

In 2022, suicide cases will recur even though the prevalence has decreased by 0.77 per cent compared to 2021, namely 30 cases, consisting of 19 men (63.33%) and 11 women (36.67%), so there is a shift in “gender quantity.” Namely, a decrease in males and females by nine people (23.07%), with livelihoods being farmers (83.33%), entrepreneurs (10%), and homemakers (6.67%). The suicide execution method used is still dominated by hanging oneself (96.67%), carried out around the house, such as in the bathroom, bedroom, kitchen, trees, and cow and goat pens. According to its type, in general, suicide by entanglement, which involves a hanger around the neck, is a typical self-hanging because the knot of the noose is located at the nape of the victim’s neck.

### 3.2 Micro dimensions of suicide in agrarian community cultures

Suicide in Gunungkidul Regency has been going on for a long time. Still, based on research, this phenomenon has begun to receive serious attention from the regional government since 2018. Suicide cases continue to occur every year, and there is even a tendency to increase in prevalence. In 2021 it rose sharply with 39 points, or 6 cases (15.38%) higher than in 2020 and 9 cases (23.07%) in 2022. Suicide cases occurred more frequently among members of rural farming families who were classified as vulnerable, such as the elderly, by hanging themselves.

Data from the Gunungkidul Regency Health Office provides an overview of the main risk factors identified in suicide cases. These factors were obtained through medical records, interviews with victims’ families, and field observations by health workers and related agencies. Table 2 summarizes the most frequently identified suicide risk factors based on this data:

#### 3.2.1 Psychological aspects of the causes of suicide

**Figure 2:**
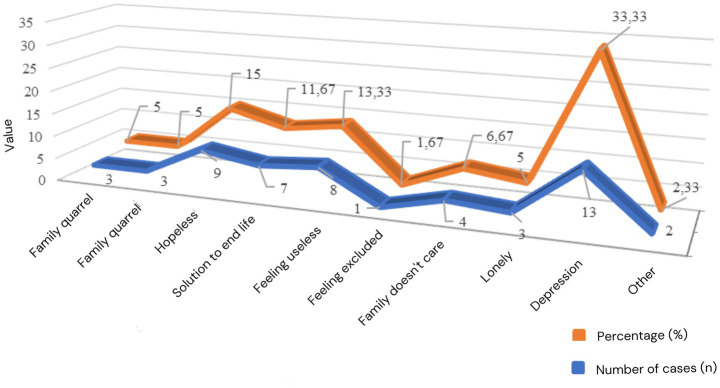
Psychological factors associated with suicide in Gunungkidul Regency.

Conditions of stress and depression are not necessarily the leading causes of suicide, but are still only precursor factors that may disrupt thinking and make a significant contribution to planning the execution of suicide. Even though it has a high risk, this precursor factor does not yet encourage someone to commit suicide. A person commits suicide when the existing precursor seeds are not balanced with personality immunity, namely the ability to be physically resilient, the mental, religious, physical, and social strength to survive and adapt to the suffering of life experienced, and then rise and fight to return to normal conditions. The more powerful the predictor factors are in an individual, the smaller the chance of committing suicide, even though the precursor factors are heavy and complex. On the other hand, the helplessness of predictor factors causes a person to have a high chance of committing the irrational act of suicide. The following describes the micro aspects of the causes of suicide in five sub-districts in Gunungkidul Regency (Figure 2).

Only one factor was found in this research: one informant (1.67%) from Semin District stated that the perpetrator felt lonely, lacked attention from family and relatives, and was ostracized by the environment due to geographical and social conditions. The perpetrator felt disconnected from his family and community. He refused to share his problems with others. This is what the following informant, speaking in Javanese, experienced: his child committed suicide.

*Informant 1:*

“Anak kulo niku mboten nate ngeluh kalih tiyang sepuh ipun, soal masalah keluarganipun dipun pendem piyambak, kok ujug ujug lajeng bunuh diri “(My child never complained to his parents, never told them about his family problems, and then suddenly committed suicide).

*Informant 2:*

“Sakjane kulo niku pun ngertos nek anak kulo gadah masalah sing abot, ning nek kulo tangletke jare ora duwe masalah opo opo, jare kancane wis nate ngeluh nek ora cocok neng nggon ne pagaweane mergo ora mampu” (I knew my child had serious problems, but when I asked him, he said he did not have any issues, and his friend said He once told me that he wasn’t suited to his job because he lacked the skills).

Observational field notes confirmed these accounts, as researchers frequently encountered elderly participants living alone in isolated hamlets, with minimal interaction from neighbors or extended family. Homes often lacked communal gathering spaces, reinforcing the theme of social withdrawal that emerged in interviews. He did not interact well within his geographical and social environment. This weak social integration gives rise to the perpetrator’s perception/ego of himself, who feels his interests and needs are ignored, so the perpetrator thinks he is not part of the family and community. The perpetrator feels like he is carrying his life alone because there are no friends/relatives/family members to release the emotions and complaints stored inside. This condition causes the perpetrator to feel isolated for a long time. Life feels depressed, hopeless, futile, and helpless socially and geographically (social and geographical isolation). Social isolation may be associated with suicide because it is an observable indicator of unmet fundamental human needs. From this social isolation, in the end, without clear reasoning, the perpetrator commits suicide.

Anomic suicidal behavior due to alienation is a personal experience as a subject who is free to have a will. This means that perpetrators who live in a social environment must have freedom and responsibility for themselves and their future, including self-harm and even suicide. From this view, researchers argue that Sartre tolerated suicide without considering that suicide incidents cannot be separated from interpersonal relations involving other people (family, relatives, society) and are contrary to social and religious norms. Ignoring suicide incidents is not a solution to the problem. Suicide will create new issues for families and society. This includes setting a bad precedent that can be used as a triggering factor and modeling suicide for other people to overcome their decision to face life’s problems.

Most of the 13 informants (families of victims) in the five sub-districts of Semin, Wonosari, Playen, Ngawen, and Karangmojo stated that the cause of the perpetrator’s suicide was due to untreated depression (33.33%), whether mild, moderate, or severe. This research found that the perpetrator’s suicide was caused by a depressive condition characterized by feelings of hopelessness, anxiety, restlessness, and loss of motivation to carry out daily activities. Hence, the perpetrator lost direction and purpose in life and ultimately ended up committing suicide. Some of the perpetrators experienced traumatic experiences related to work due to family economic problems, marriage, and family disputes that lasted for a long time without resolution.

There were nine informants (15%) who stated that there was a feeling of hopelessness and boredom with life, which contributed to the perpetrator taking his own life. Feelings of distress can be interpreted as an inner condition that gives up and admits defeat in overcoming external problems. The perpetrator’s poor internal condition develops into feelings of pessimism about his life, leading to thoughts, plans, and suicide attempts. For individuals like those above who do not have strong personality immunity, the impasse in overcoming problems is considered psychologically a hefty burden that drives the perpetrator to commit suicide. Eight informants (13.33%) stated that the perpetrator felt useless to the family and society. Next, in a row, seven people (11.67%) declared suicide as suicide as a solution to life. Emile Durkheim called it an anomic act of suicide. Family is not cared for (6.67%); Family quarrels and feeling lonely due to the death of a partner were experienced by three people each (5.0%). The remaining two people (2.33%) were due to the perpetrator’s desire to marry, which the family rejected, and failure to pursue spiritual knowledge.

Depression and anxiety were notably prevalent among victims, primarily influenced by the dual burden of economic stress and chronic illness (Zafar et al., 2024). Economic hardships, often exacerbated by limited access to financial resources or employment opportunities, created instability and uncertainty (Nasr et al., 2024). Simultaneously, chronic illnesses, whether pre-existing or triggered by prolonged exposure to adverse conditions, intensify psychological distress by adding physical limitations and healthcare costs to an already challenging situation (Bryant, 2019). This interplay between financial insecurity and health-related challenges underscores the multifaceted nature of mental health struggles in vulnerable populations, emphasizing the urgent need for integrated support systems that address both economic and health dimensions to alleviate psychological burdens.

#### 3.2.2 Economic aspects of the causes of suicide

**Figure 3:**
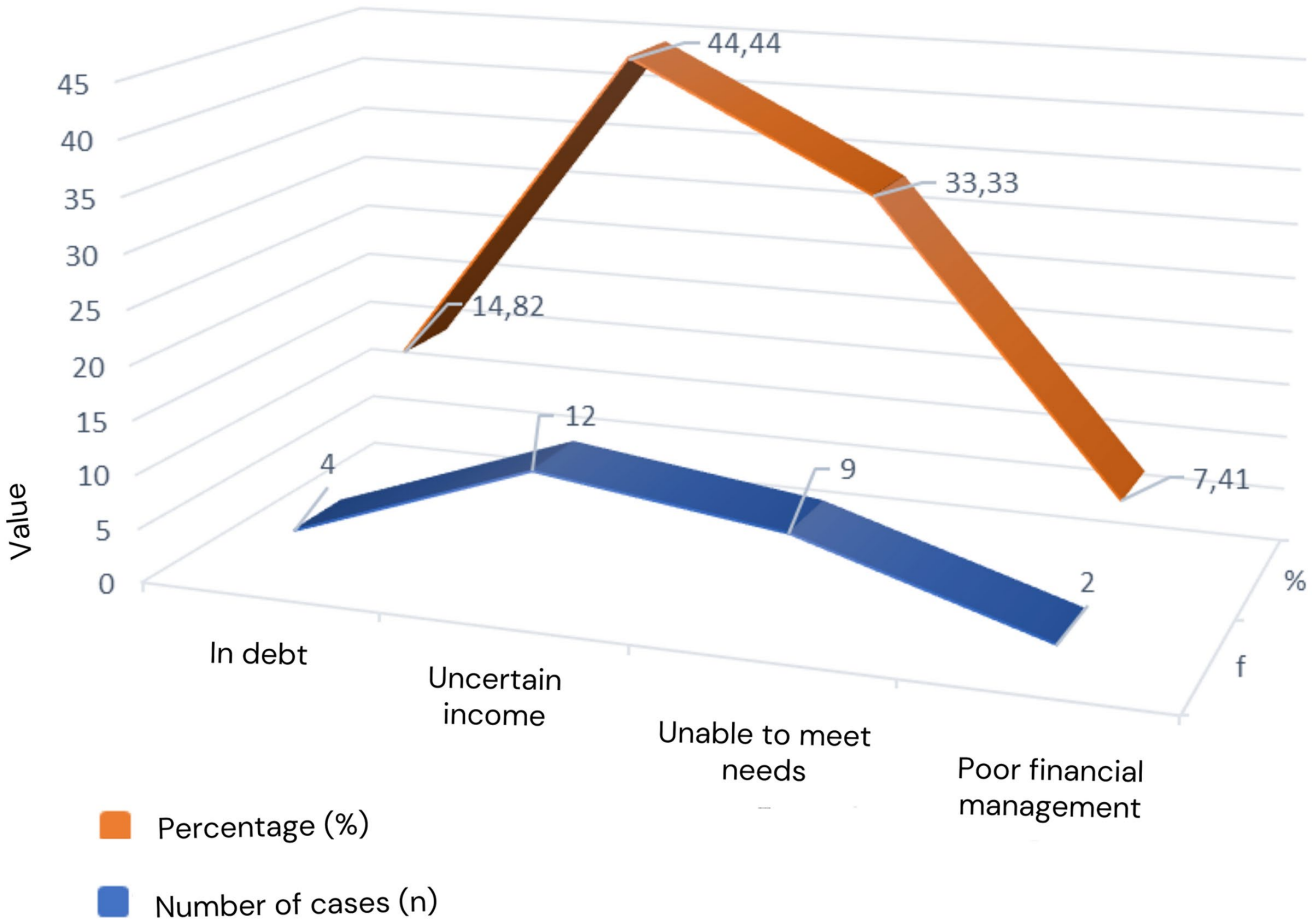
Economic factors associated with suicide in Gunungkidul Regency.

In the culture of pastoral communities in rural areas in Gunungkidul, the agricultural sector is synonymous with the main livelihood. For the most part, agricultural communities in rural areas are farming entities that can fulfil their basic needs economically through farming in paddy fields and fields (Figure 3).

However, crop failures, lean seasons, long dry seasons, and other factors make them powerless to meet the family’s adequate needs. Their income became unstable (44.44%), decreasing drastically, so they could not meet their living needs (33.33%). Even to meet their daily needs, some are forced into debt with loan sharks (14.82%) and experience problems in managing family finances (7.41%). Various issues that are not resolved can slowly cause feelings of anxiety, stress, and psychological disorders such as depression, and can even cause mental health problems. Mental health disorders due to family economic problems can, in the long term, lead to behavioral effects that society considers abnormal behavior, namely, committing suicide.

Based on the results of interviews and observations at the research location, it was revealed that in the culture of the agricultural community in rural Gunungkidul, multiple micro factors were found to cause precursors to suicide in the economic aspect, where the suicide rate was committed mainly by older adults, namely 11 people (73, 33%). According to gender, the suicide rate for men is higher than for women, with details of 12 men (80%) and three women (20%). The burden of responsibilities in the family, poverty, and the decreasing role of the economy in the patriarchal culture of agrarian society cause men as heads of families to be more vulnerable to mental problems, including depression. This situation negatively impacts the emergence of ideas and plans and can lead to suicide. The following informant stated that economic reasons led to suicide, including job loss.

*Informant 3:*

“Bubar kecalan pedamelan, nyebabaken dipegalih saestu lan grantes nimbulaken putus asa, sering ngalamun, pikirane grambyang, ngengleng akhiripun nglalu bunuh diri.” (After losing a job, it causes heavy thoughts that lead to despair, daydreaming, wandering thoughts, and dizziness, which ultimately leads to suicide).

*Informant 4:*

“Prekara ing pikiran abot amorgo ora iso nyukupi kebutuhan keluarga, nyebabke nglokro sing ngakibatke bunuh diri” (The problem of thinking too hard about not being able to meet the family’s needs, which results in suicide).

During site visits, researchers observed barren fields and dry riverbeds in several sub-districts during the prolonged dry season, which community members linked to crop failures. Field notes described conversations among farmers expressing worry about repaying debts to local lenders, underscoring the financial instability reported by interviewees. Suicide in rural and agrarian communities often arises from a complex interaction of interrelated challenges that exacerbate individual vulnerabilities, creating a cyclical relationship between economic stress and psychological despair. Financial instability, driven by fluctuating income sources or unemployment, undermines the ability to meet basic needs and familial obligations. Similarly, debt frequently incurred to finance agricultural inputs or other essential expenses becomes unmanageable when compounded by high interest rates and unpredictable repayment conditions. Crop failures caused by climatic variability, pest infestations, or limited resource access further intensify these pressures by eroding primary livelihoods. Collectively, these factors form formidable barriers to resilience, disproportionately impacting marginalized populations. Addressing these issues demands urgent policy interventions targeting such distress’s structural and systemic roots.

Qualitative data from interviews show that suicides in Gunungkidul Regency tend to increase during the dry season. For many residents who depend on agriculture for their livelihood, crop failure means the loss of their primary source of income. This situation is characterized by a food shortage due to crop failure. Severe economic pressure, coupled with a sense of hopelessness due to the lack of alternative livelihoods, triggers mental health disorders. The following is a statement from a village head whose resident committed suicide.

In our study, hanging was the most common method of suicide (97.4%), with individuals often using plastic ropes tied around the neck. These deaths typically occurred in domestic spaces such as bedrooms, kitchens, bathrooms, or nearby structures like trees and animal pens (Table 3). Poison ingestion (2.6%) and drowning in wells were rarely reported. This pattern is consistent with published studies in other agrarian contexts, where hanging is also the predominant method of suicide, followed by pesticide ingestion, largely due to its availability in farming communities (Arafat et al., 2021; Nagar et al., 2022; Stack, 2021).

**Table 3:** Distribution of suicide methods, locations, and demographic patterns of victims.

Variable	Category	*n*	% of total cases
Gender	Male	25	64.10
	Female	14	36.15
Age group (years)	60–91	28	71.79
	36–57	9	23.07
	15–35	2	5.14
Occupation	Farmer/Farm laborer	32	82.05
	Homemaker	3	7.69
	Student	2	5.13
	Fisherman	1	2.56
	Casual laborer	1	2.56
Method	Hanging (rope, plastic rope, sling, sarong, scarf, belt)	38	97.44
	Poison ingestion	1	2.56
Location	Home (inside)	22	56.41
	Tree	8	20.51
	Animal cage	6	15.38
	Hut	1	2.56
	Farm	1	2.56
	Drying area	1	2.56
Reported contributing factors	Depression, economic hardship, chronic illness, loneliness, family conflict, cultural beliefs (pulung gantung)	—	—

*Informant 5.*

“Saat hasil panen gagal akibat musim paceklik (maksudnya kemarau berkepanjangan), banyak warga petani kehilangan pendapatan utama. Rasa terhimpit dan tidak tahu harus berbuat apa seringkali menimbulkan stres berat yang berdampak ke kondisi mental, bahkan tindakan bunuh diri (“When crops fail due to the dry season (meaning a prolonged drought), many farmers lose their primary source of income. Feelings of being squeezed and unsure of what to do often lead to severe stress, which can lead to mental health issues and even suicide”).

The prolonged dry season also creates increasingly difficult environmental conditions: water shortages, barren land, and difficulty meeting daily needs. These conditions exacerbate the mental burden, especially for those without access to social support. In Gunungkidul Regency, the stigma surrounding mental health issues remains very strong. Many people stay silent and suppress their feelings for fear of being perceived as weak or embarrassed to speak up.

#### 3.2.3 Physical health aspects cause suicide

**Figure 4:**
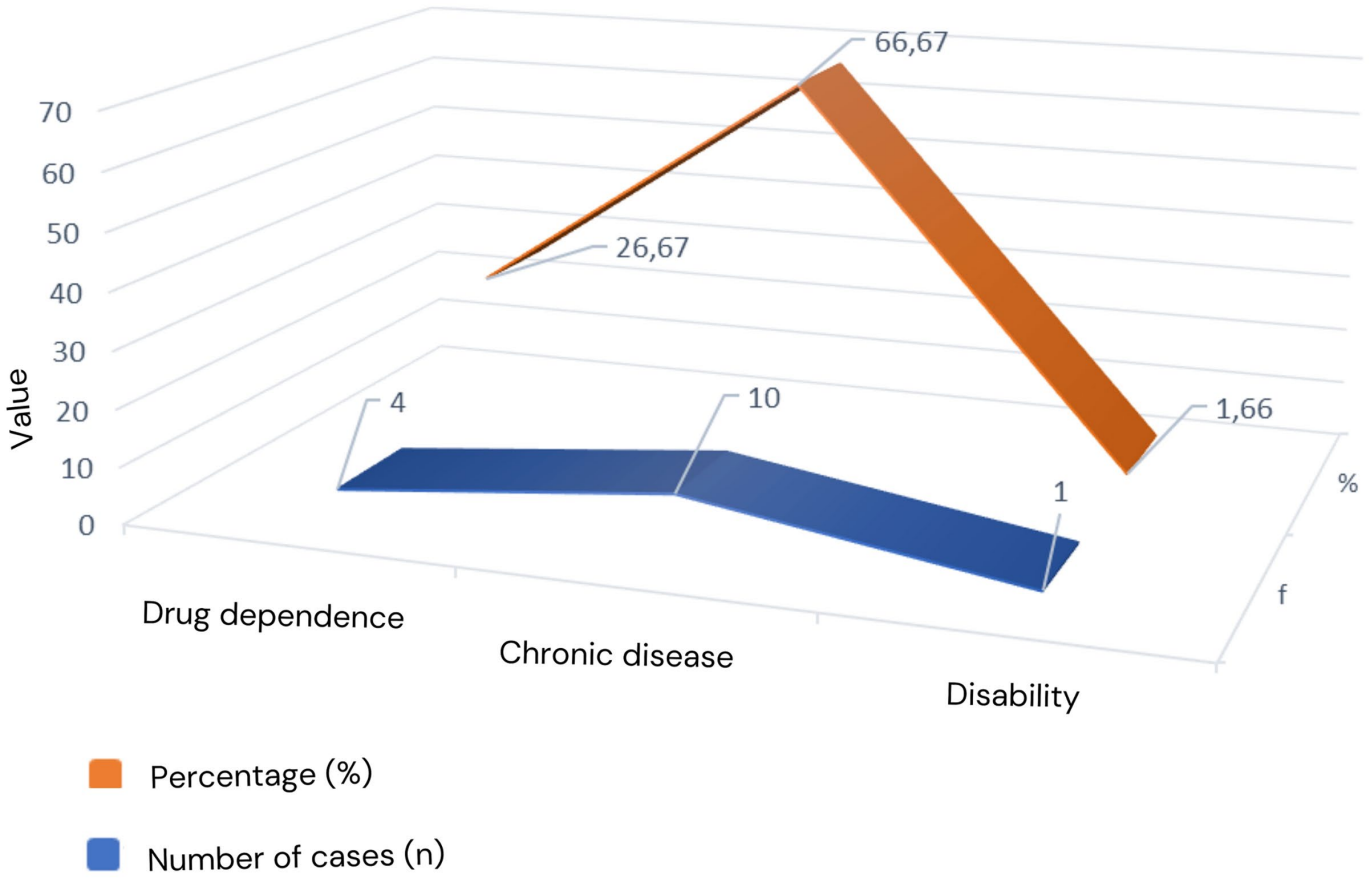
Physical health factors causing suicide.

Based on information from the victim’s family, the suicide victims were spread across five sub-districts in Gunungkidul Regency. The perpetrator had a history of drug dependence and health problems in the form of illnesses that caused him to be disabled. Figure 4.

This study identified three physical health conditions as risk factors that could lead to increased suicide. Based on information from informants, it was found that three perpetrators in Playen District, three perpetrators in Wonosari District, and four perpetrators in Samin District had chronic health problems. In Ngawen District, four people committed suicide due to drug addiction. The remaining perpetrators in Karangmojo District had disabilities due to diabetes.

*Informant 6:*

“Mergo penyakite ora mari mari deweke rumongso membebani keluarga pungkasane mutuske ngakhiri urip kanti bunuh diri” (Because his illness did not get better, he felt he was a burden on his family and finally decided to end his life by committing suicide).

*Informant 7:*

“Kerep mlebu metu rumah sakit ning lorone ora mari, piyambakipun rumaos membeni keluarga pungkasane ngetokake putusan nglalu (He was often hospitalized, but his illness did not get better. He felt he was a burden on his family, and finally committed suicide).

Observational notes taken during visits to households of people with chronic illness highlighted limited access to health facilities and the visible burden placed on family caregivers. These observations aligned with participants’ descriptions of physical suffering, dependency, and feelings of being a burden, which contributed to suicidal distress. The act of suicide to end life was carried out by 15 victims in five sub-districts in Gunungkidul Regency, 14 of whom committed suicide due to depression. The perpetrator feels hopeless. There is no hope and no other alternative to overcome psychological and economic dilemmas and severe physical disorders in his life. The remaining person committed suicide due to depression and psychological disorders in the form of hallucinations, where the perpetrator felt subtle whispers were ordering him to commit suicide. The method most often used to achieve suicide was hanging oneself. There were 13 victims (87.66%), one person throwing themselves into a well (6.67%), and one person drinking potassium poison (6.67%).

The Gunungkidul Regency government plays a crucial role in preventing suicides due to mental health disorders by providing affordable services. By providing counseling facilities at community health centers, training health workers, and educating the community about the importance of maintaining mental health, the local government strives to reduce stigma and raise awareness. The sub-district head stated the following:

*Informant 8:*

“Kami menyadari bahwa gagal panen bukan hanya soal kerugian ekonomi, tapi juga bisa berdampak pada kesehatan mental. Oleh karena itu, pemerintah daerah terus berupaya memberikan bantuan seperti subsidi benih, distribusi pangan, dan layanan konseling agar masyarakat tetap kuat menghadapi tantangan.” (We realize that crop failure is not only about economic losses, but can also impact mental health. Therefore, the local government continues to provide assistance such as seed subsidies, food distribution, and counseling services to help the community remain resilient in facing challenges).

In addition, social and economic assistance is provided to vulnerable groups, especially during crises such as crop failure or natural disasters, which can trigger severe psychological distress. The local government also forms a cross-sector network—involving community leaders, health workers, and regional organizations—to detect early risks and respond rapidly to needy residents. With these steps, the local government affirms its commitment to protecting the mental health of the community and preventing the loss of life due to despair.

## 4 Discussion

In this study, two social constructs were identified as related to suicide occurring in the culture of an agrarian society in rural Gunungkidul, namely altruistic and egoistic motives (Khan and Arendse, 2023). Altruistic motives were observed in communities with strong social integration, where individuals, particularly men as family breadwinners, felt deep psychological burdens when unable to fulfill their responsibilities (Ilzam, 2019). Egoistic motives were associated with weakened social cohesion, where individuals felt socially isolated and disconnected from community and family support systems (Moyano et al., 2022).

In the patriarchal construction, men are placed as heads of families who are fully responsible as the family’s primary breadwinners. The perpetrator’s inability to meet the family’s needs causes psychological burdens (Ekberg et al., 2002). The perpetrator is aware, embarrassed, and depressed about his failure to adapt to the socio-economic demands of family and society. Starting from fragile mental resilience (Zou et al., 2022), this condition encourages irrational actions to the point of ending life.

From a cultural perspective, the myth of pulung gantung illustrates how strong social integration and traditional beliefs can shape community perceptions of suicide. This belief, where a reddish light is thought to foreshadow a suicide in the household it falls upon, was reported in Playen, Wonosari, and Karangmojo sub-districts (Mursidi and Noviandari, 2021). Within this cultural construction, individuals who died by suicide were sometimes perceived as acting in accordance with cultural or spiritual phenomena, rather than solely personal distress.

The number of suicides in Gunungkidul is also inseparable from the weakening of social integration in society, where individuals are less obedient and even tend to ignore the norms, values, and regulations adopted by the community. Regional geography is thought to alienate perpetrators due to a lack of concern from family and society over time (Moyano et al., 2022). In conditions of solitude, without relatives, family, and friends, the perpetrator fails to release emotions (catharsis), complains, and various problems to relieve the socio-economic issues they face (Lyu et al., 2025). All life’s problems are borne by yourself. At the same time, the weakening of social integration contributed to egoistic suicides. Individuals who lived in solitude, with limited concern or support from family and society, experienced heightened vulnerability. In such cases, persons who felt isolated carried the burden of life problems alone, without avenues for catharsis or social release. Depression, loneliness, and alienation ultimately contributed to decisions to end life. This type of suicide occurs more often in Semin and Ngawen Districts.

This study further highlighted three interrelated micro-level factors: psychological distress, economic hardship, and chronic health conditions that were associated with suicide in Gunungkidul. These factors, often unrecognized or “hidden” within daily life, shaped individual vulnerability to suicidal behavior.

- Psychological distress included untreated depression, feelings of hopelessness, social isolation, and unresolved family conflict.
- Economic hardship refers to crop failures, unstable income, indebtedness to local lenders, and difficulty meeting household needs.
- Chronic health conditions encompassed physical illness, disability, and long-term treatment burdens that left individuals feeling like a burden to their families.

Rather than functioning in isolation, these factors interacted to intensify despair. For example, economic instability often worsens depression, while illness compounds both financial strain and psychological suffering. Observational data reinforced these findings, showing barren fields during the dry season, limited access to healthcare, and the visible strain placed on caregivers in households affected by chronic illness.

In the micro dimension, research in Gunungkidul Regency found hidden factors that are interrelated as causes of suicide. These factors are the initial factors, triggers, or raw materials that make a person have a high potential for developing suicidal ideas and plans. These factors are psychological, economic, and physical health factors. Aspects of psychological disorders (Moitra et al., 2021) include traumatic experiences, the death of a partner, loneliness, and other complex problems that have no solution, which can cause anxiety, feelings of hopelessness, inner pressure, stress, and depression. If internal conditions are not managed well, it can become a raw material for suicide. These natural ingredients can trigger thoughts, ideas, and plans that lead to the possibility of committing suicide, but do not always end in extreme actions in the form of suicide.

Economic factors (Stack, 2021) and physical health problems also have a high risk of committing suicide as long as the perpetrator has weak elements such as mental health, personality, immunity, and spirituality. This factor is the leading cause of suicide or, conversely, can prevent someone from committing suicide. The actions of suicide victims show weak mental health and spirituality. The most common way victims commit suicide is by hanging themselves using a plastic rope tied around their neck, causing death. Suicide locations include houses, trees, and animal pens. Hanging is the most common conventional method of suicide in the culture of rural farming communities in Gunungkidul. They were followed by using poisonous pesticides and drowning themselves by jumping into wells.

The local government provides clinical psychologists with mental and physical health screening tests to detect residents who are indicated to be experiencing psychological and physical health problems early (Ibrahim et al., 2019). Establishment of independent cooperatives for residents experiencing economic constraints. Guidance and counselling for families about suicide through religious harmony forums and activities in the community. “Early detection of mental health disorders plays a vital role in suicide cases.” Suicide cases do not appear suddenly, but mental disorders can be detected early with psychiatric screening tests for people experiencing health problems. Early detection occurs (Kusumoningrum et al., 2025). Formation of independent cooperatives as an alternative forum for communities in need to support the family’s economic life (Panesar et al., 2021). They provide food assistance to needy people to reduce the financial burden on families.

Chronic illness and disability significantly increase the risk of suicidal ideation, as they often contribute to emotional, psychological, and social burdens (Zhang et al., 2025). Individuals living with chronic conditions or disabilities frequently face challenges such as persistent physical pain, limited mobility, social isolation, and stigmatization, all of which can exacerbate feelings of hopelessness and depression (Wang et al., 2021). These factors not only strain mental health but also diminish perceived quality of life, thereby elevating vulnerability to suicidal thoughts. Understanding this heightened risk is crucial for developing targeted mental health interventions and policies aimed at providing comprehensive support for affected individuals, reducing stigma, and improving access to mental health resources (Carpiniello and Pinna, 2017).

A study in North India revealed that the majority of suicides were committed by young men aged 20–29, with hanging as the primary method, and cases peaked during the summer (April–June). This study emphasized the importance of age, gender, method, and season as key factors in understanding suicide patterns (Nagar et al., 2022). Compared to Gunungkidul Regency, hanging was also the most common method. However, demographically, suicide victims in Gunungkidul were more likely to be older, particularly those aged 60 and above, and most worked as farmers. This suggests that the burden of social, economic, and health factors is significant, especially among older people.

Explicitly analyzing seasonal variations in Gunungkidul, the geographical and agrarian context makes it particularly vulnerable to suicide during the prolonged dry season. This is also exacerbated by climate change, which causes crop failures, leading to economic stress. These factors are suspected to contribute to increased suicide rates at certain times of the year, similar to the seasonal patterns identified in North India. Based on the Indian study’s approach, it can be concluded that suicide prevention efforts in Gunungkidul need to consider unique local characteristics, such as the vulnerability of the elderly, the stress of life in the agricultural sector, and the potential impact of the dry season on mental health. This demographic and seasonal data-driven approach is crucial for designing more targeted and contextual interventions.

Understanding suicide in rural agrarian contexts requires attention to both cultural constructions and micro-level vulnerabilities. Efforts to reduce suicide must therefore combine early detection of mental health concerns, economic empowerment strategies such as cooperatives, and community education to challenge persistent myths. Interventions should be person-centered, reducing stigma while strengthening family and community support networks to protect individuals at risk of suicidal distress.

## 5 Conclusion

This study concludes that the interaction of cultural constructs and micro-level vulnerabilities shapes suicide in rural agrarian communities. Two broad hidden dimensions are identified: first, sociocultural constructs encompassing altruistic and egoistic motives influenced by strong or weak social integration, local myths such as pulung gantung (hanging), and patriarchal family expectations. Second, micro-level factors include psychological distress, economic hardship, and chronic health conditions. These everyday issues often remain unnoticed until they accumulate, creating a high potential for developing suicidal thoughts, plans, and, ultimately, suicidal acts when combined with weak protective factors such as mental resilience, social support, and spirituality.

Seasonal challenges, particularly crop failures during prolonged droughts, further exacerbate these risks by exacerbating economic and mental stress. Effective prevention requires early detection of psychological distress, economic empowerment through cooperatives and social safety nets, increased access to health services, and culturally sensitive education to dispel persistent myths. Therefore, suicide prevention in Gunungkidul must be individual-centered, stigma-reducing, and community-based, integrating family, cultural, and structural interventions.

## Data Availability

The raw data supporting the conclusions of this article will be made available by the authors, without undue reservation.
